# Swertiajaponin inhibits skin pigmentation by dual mechanisms to suppress tyrosinase

**DOI:** 10.18632/oncotarget.20913

**Published:** 2017-09-15

**Authors:** Bonggi Lee, Kyoung Mi Moon, Bong-Seon Lee, Ju-Hye Yang, Kwang Il Park, Won-Kyung Cho, Jin Yeul Ma

**Affiliations:** ^1^ Korean Medicine (KM)-Application Center, Korea Institute of Oriental Medicine (KIOM), Daegu 41062, Republic of Korea

**Keywords:** swertiajaponin, melanogenesis, pigmentation, human skin, tyrosinase

## Abstract

Many skin-whitening compounds target tyrosinase because it catalyzes two rate-limiting steps in melanin synthesis. Although many tyrosinase inhibitors are currently available for a skin–whitening purpose, undesirable adverse effects are also reported. Thus, numerous efforts have been made to develop safer tyrosinase inhibitors from natural products. In line with this, we tested fifty flavonoids, a group of naturally occurring antioxidants and metal chelators, and screened swertiajaponin as the strongest tyrosinase inhibitor in cell-free experiments. Swertiajaponin did not show cytotoxicity in B16F10, HaCat, and Hs27 cells and exhibited strong anti oxidative activity in experiments using the cell-free system and B16F10 cells. It markedly inhibited αMSH- or UVB-induced melanin accumulation in B16F10 cells and suppressed skin pigmentation in a human skin model. As underlying mechanisms, in silico and Lineweaver-Burk plot analyses exhibited that swertiajaponin may directly bind to and inhibit tyrosinase activity by forming multiple hydrogen bonds and hydrophobic interactions with the binding pocket of tyrosinase. In addition, western blotting results indicated that swertiajaponin inhibited oxidative stress-mediated MAPK/MITF signaling, leading to decrease in tyrosinase protein level. Together, swertiajaponin suppresses melanin accumulation by inhibiting both activity and protein expression levels of tyrosinase. Thus, it would be a novel additive for whitening cosmetics.

## INTRODUCTION

Skin is a major site where melanin pigments are accumulated. Melanin synthesis and accumulation occur as a protective mechanism against ultraviolet (UV) irradiation. Thus, people with black skin are at lower risk for skin cancers than those with pale skin because they are protected from the mutagenic effects of UV [1]. However, uncontrolled melanogenesis is closely associated with pigmentation disorders including melisma, freckles, and senile lentigines [2]. In addition, abnormal alterations in skin color result in unfavorable cosmetic problems and eventually degrade the quality of life. Thus, the prevention of abnormal melanogenesis has attracted substantial attention for the development of skin-whitening cosmetics [3–5].

The melanocyte is the main cell type that synthesizes melanin in the epidermis of the skin. After synthesis, melanocytes transfer melanin to keratinocytes through the dendrites, leading to skin pigmentation. Melanin can be synthesized from L-tyrosine through hydroxylation and oxidation processes, in which a multifunctional copper-containing tyrosinase plays a pivotal role in melanin synthesis. Tyrosinase catalyzes two rate-limiting steps in melanin synthesis; the monophenolase hydroxylation of L-tyrosine to 3,4-dihydroxy-L-phenylalanine (L-DOPA), and the oxidation of L-DOPA to DOPA quinine [6, 7]. Accordingly, tyrosinase inhibition is a good strategy to brighten the skin, but cytotoxicity and lack of stability have limited the application of various tyrosinase inhibitors in the field of medical products and cosmetics. Thus, unceasing efforts have been made to find safer tyrosinase inhibitors with stronger stability and selectivity from natural sources.

As an effort to find compounds for better and safer whitening effect for skin, we tested fifty flavonoids available naturally for their inhibitory effects on tyrosinase and screened swertiajaponin as the strongest tyrosinase inhibitor. We further investigated its effect on skin pigmentation using cell and human skin models with various biological assays and *in silico* analysis.

## RESULTS AND DISCUSSION

### Swertiajaponin is the strongest tyrosinase inhibitor of fifty flavonoids

**Figure 1:**
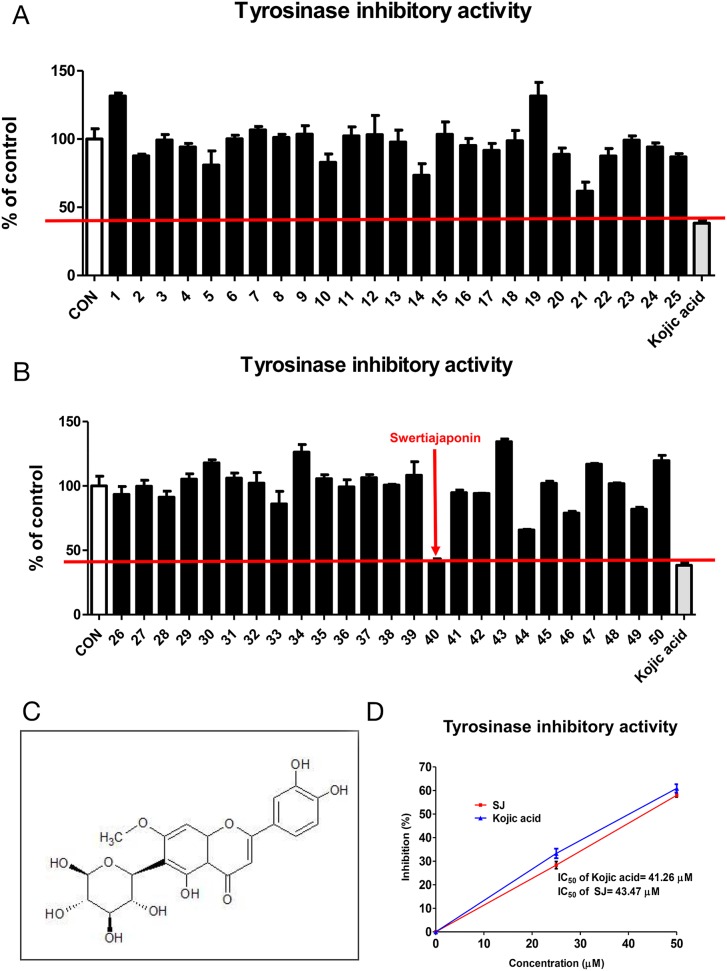
Swertiajaponin is the strongest tyrosinase inhibitors of fifty flavonoids **(A-B)** The tyrosinase inhibitory activities of fifty flavonoids were measured using mushroom tyrosinase and L-tyrosine as a substrate. The inhibition percentage of kojic acid, a positive control, was used as selection criteria. **(C)** The structure of swertiajaponin was drawn using the ChemSketch software. **(D)** The inhibitory concentration 50% (IC_50_) of swertiajaponin and kojic acid was determined in the cell-free experiment using mushroom tyrosinase and L-tyrosine (n=3).

Of various natural compounds, flavonoids, a group of naturally occurring antioxidants and metal chelators, have been known to suppress tyrosinase activity because of their ability to form copper-flavonoid complexes [8, 9]. We used fifty flavonoids that were commercially available to test whether they have inhibitory activity against mushroom tyrosinase. Kojic acid, a well-known tyrosinase inhibitor, was used as a positive control to screen better tyrosinase inhibitors (Figure 1A-1B). As a result, sample number 40 (swertiajaponin) (Figure 1C) exhibited the strongest inhibitory activity against tyrosinase than that of other flavonoids (Figure 1A-1B and Supplementary Figure 1). When the inhibitory activity was further examined by a concentration-dependent experiment, the IC_50_ value of kojic acid was 41.26 μM and that of swertiajaponin was 43.47 μM (Figure 1D), indicating that tyrosinase activity inhibition of swertiajaponin is comparable to that of kojic acid based on test tube experiments.

### Swertiajaponin shows no cytotoxicity *in vitro*

**Figure 2:**
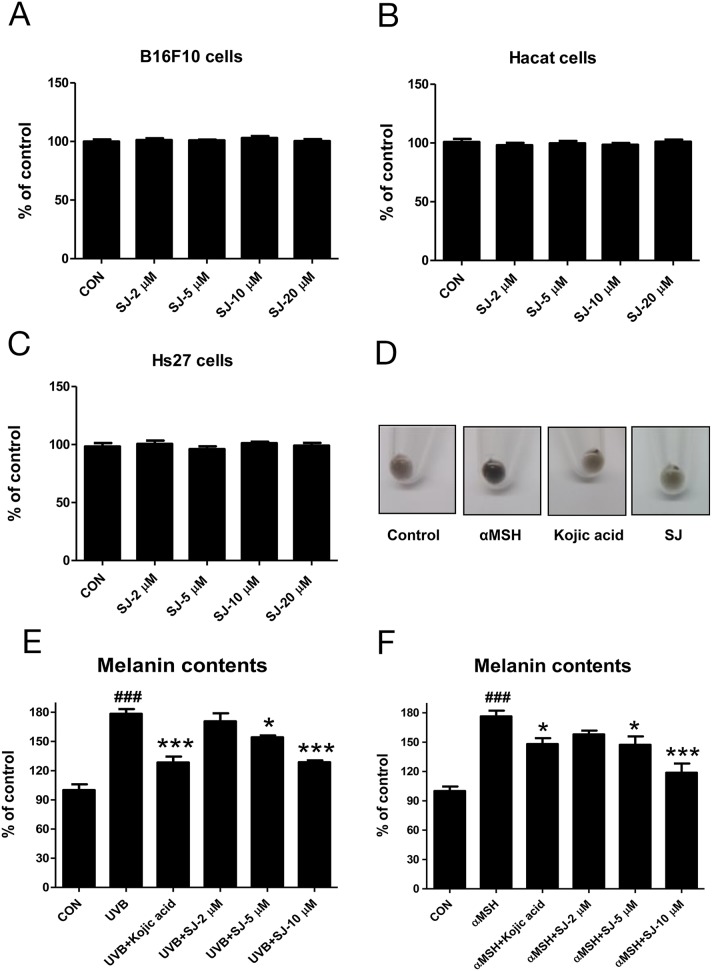
Swertiajaponin inhibits melanin level in B16F10 cells Cell viability was examined in **(A)** B16F10, **(B)** HaCat, and **(C)** Hs27 cells treated with various doses of swertiajaponin (2-20 μM) for 48 h. The cytotoxicity was examined using a commercially available assay system (see methods for the detailed information). The data was shown as the percentages relative to the control group (non-treated group) (n= 3/group). B16F10 cells were pretreated with swertiajaponin or kojic acid for 1 h and the cells were stimulated with **(D-E)** αMSH (500 nM) or **(D)** UVB (10 mJ) for additional 48 h (n=4/group) with swertiajaponin or kojic acid treatment. The data are expressed as means ± SEM. Data were analyzed using one-way ANOVA followed by Dunnett's test. ^###^P < 0.05 compared with the non-treated control group. ^*^P < 0.05, ^**^P < 0.01, and ^***^P < 0.001 compared to the α-MSH or UVB-treated group.

Swertiajaponin belongs to the family of flavonoid C-glycosides. It contains a carbohydrate moiety which is C-glycosidically linked to one of the flavonoid backbones (Figure 1C). It has been reported as a compound found in the whole herb of the Swertia japonica [10]. Although some studies reported that the Swertia japonica is used clinically as a remedy for gastrointestinal symptoms in Japan [11], few studies are available about biological functions of swertiajaponin. Thus, we further examined the effects of it on melanogenesis. Because cytotoxicity is a major issue of synthetic tyrosinase inhibitors, we tested whether swertiajaponin showed cellular toxicity using multiple cell lines including HaCat (human keratinocyte), B16F10 (mouse melanoma), and Hs27 (human fibroblast). Swertiajaponin showed no cytotoxicity in these cell lines (Figure 2A-2C).

### Swertiajaponin inhibits melanogenesis in B16F10 cells

We studied its effect on melanogenesis using B16F10 cells pre-treated with kojic acid or swertiajaponin followed by UVB or αMSH exposure, the strongest melanogenic inducers. As expected, UVB or αMSH exposure notably increased melanin level in B16F10 cells (Figure 2D-2F) and swertiajaponin treatment reduced cellular melanin content in a dose-dependent manner post UVB or αMSH exposure (Figure 2D-2F). In cell studies, the anti-melanogenic effect of swertiajaponin appears to be stronger than that of kojic acid (Figure 2E-2F). Similarly, the color of the cell pellet pre-treated with swertiajaponin before αMSH treatment was brightened compared to the αMSH-treated cells (Figure 2D), indicating that swertiajaponin inhibits UVB or αMSH-induced melanogenesis.

### Swertiajaponin inhibits melanogenesis in a human skin model

**Figure 3:**
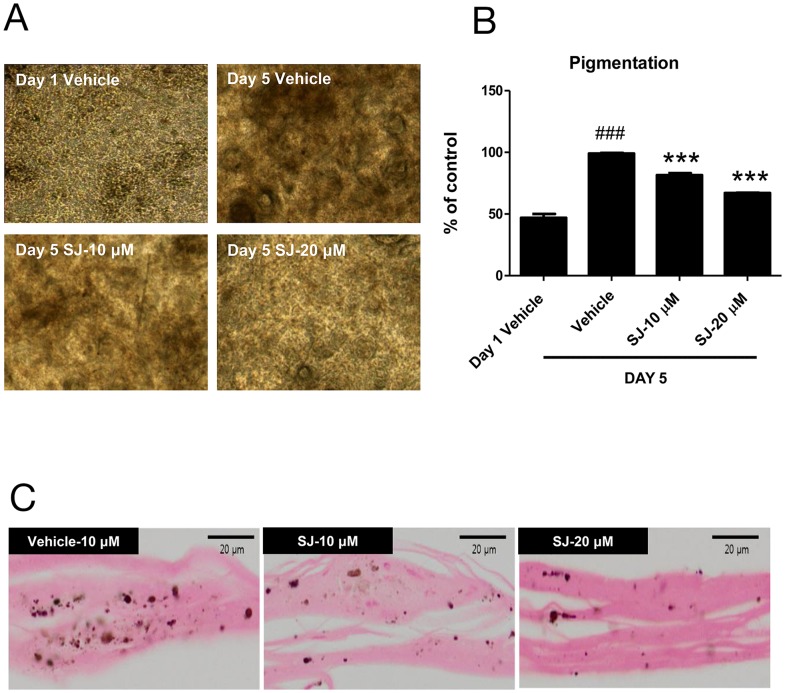
Swertiajaponin suppresses melanin accumulation in a human skin model The human skin model was treated with DMSO or swertiajaponin (10-20 μM) in the maintenance media provided by the company for 5 days. **(A)** Representative microscopic images of the human skin model with or without swertiajaponin treatment. **(B)** Changes in the darkness values of the skin were analyzed by Image J software. **(C)** Fontana-Masson staining showing the epidermis of human skin section with or without swertiajaponin treatment. Each value was expressed as mean ± SEM. ^###^P < 0.05 compared with the day 1 group without any treatment, ^***^P < 0.001 compared to the day 5 group.

To confirm that swertiajaponin suppresses skin pigmentation in human, we cultured a viable, three-dimensional, reconstituted human epidermis consisting of human melanocytes and keratinocytes that have been known to undergo spontaneous melanogenesis with time [12]. We pretreated the human skin model with swertiajaponin for 1 h and cultured it in the maintenance medium for 5 d. Compared to the day 1, the human skin model was darkened at day 5 and swertiajaponin treatment prevented it in a concentration-dependent manner (Figure 3A-3B). Similarly, Fontana-Masson staining exhibited that swertiajaponin markedly reduced melanin level in the epidermis (Figure 2C), suggesting that swertiajaponin decreases pigmentation in the human skin model.

### Swertiajaponin may bind to and inactivate tyrosinase

**Figure 4:**
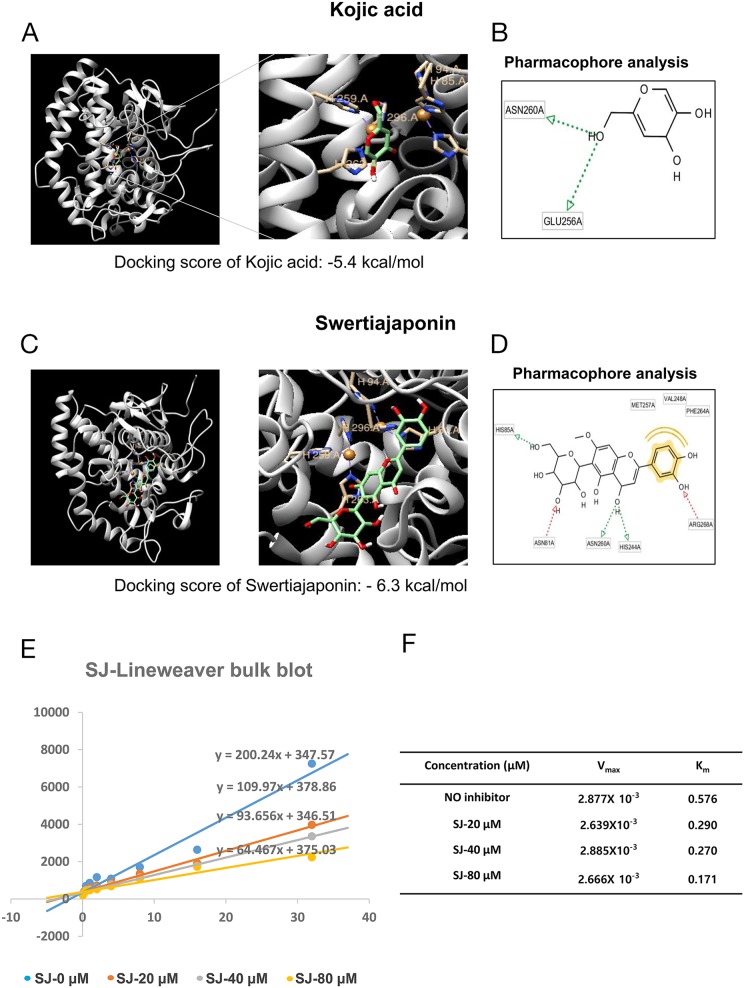
Swertiajaponin is a competitive inhibitor of tyrosinase The binding affinity of **(A)** kojic acid or **(C)** swertiajaponin with tyrosinase (Agaricus bisporus, PDB ID:2Y9X) predicted by protein docking simulation using AutoDock Vina. LigandScout 3.1 software was applied to investigate binding residues of tyrosinase that interact with **(B)** kojic acid or **(D)** swertiajaponin. **(E)** The Lineweaver-Burk plot analysis was done to examine the mode of inhibition by swertiajaponin. **(F)** Km is the Michaelis–Menten constant and Vmax is the maximum reaction velocity based on Lineweaver–Burk plot analysis.

We next investigated mechanisms underlying the swertiajaponin-mediated anti-melanogenic effects using B16F10 cells. Because swertiajaponin suppressed tyrosinase activity in the cell-free experiments using mushroom tyrosinase (Figure 1B), we hypothesized that it directly binds to and deactivate tyrosinase. To test this, protein-ligand docking simulation was performed to predict binding affinity using AutoDock Vina. The predicted binding affinity between tyrosinase and kojic acid was -5.4 kcal/mol and the affinity between tyrosinase and swertiajaponin was -6.3 kcal/mol (Figure 4A and 4C), suggesting that swertiajaponin may bind to tyrosinase with stronger affinity than kojic acid does. To further investigate the binding mode between compounds and tyrosinase, pharmacophore analysis was performed using LigandScout 3.1 software. While kojic acid may bind to tyrosinase mainly via hydrogen bonds with the ASN260 or GLU256 residue of tyrosinase (Figure 4B), swertiajaponin forms multiple hydrogen bonds with HIS85, ASN81, ASN260, HIS244, and ARG268 residues of tyrosinase and hydrophobic interactions with MET257, VAL248, and PHE264 residue of tyrosinase (Figure 4D), which likely explains the higher binding affinity of swertiajaponin to tyrosinase.

Because a predefined binding site of tyrosine was applied as a docking pocket in the protein-ligand docking simulation [3], it is likely that swertiajaponin may competitively bind to tyrosinase with tyrosine. To examine this, a Lineweaver-Burk analysis was performed. As the concentration of swertiajaponin elevated, K_m_ values also increased while V_max_ values were consistent (Figure 4E-4F), indicating that swertiajaponin is a competitive inhibitor of tyrosinase.

### Swertiajaponin is an antioxidant that suppresses UVB-induced MITF/tyrosinase signaling

**Figure 5:**
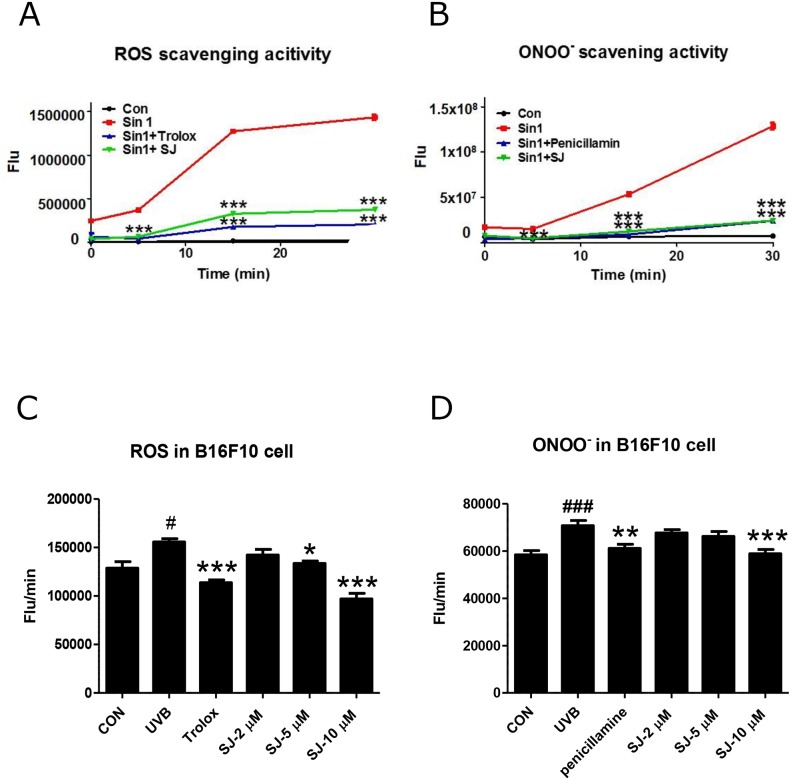
Swertiajaponin reduces UVB-induced oxidative stress in B16F10 cells To test the anti oxidative effect of swertiajaponin, generation of **(A)** ROS and **(B)** ONOO- was measured in a cell-free system using fluorescent probes DCFDA and DHR123, respectively. Sin1 was used as a stimulator for ROS and ONOO- generation and Trolox and penicillamine were used as positive controls to scavenge ROS and ONOO-, respectively. **(C-D)** To investigate the anti oxidative function of swertiajaponin in B16F10 cells, the cells were pretreated with swertiajaponin (2-10 μM) for 1 h, followed by UVB exposure (10 mJ) for 30 min. Inhibition of intracellular levels of **(C)** ROS and **(D)** ONOO- are shown. The data are shown as the mean ± SEM. Data are analyzed using one-way ANOVA followed by Dunnett's test: ^#^P < 0.05 and ^###^P < 0.001 compared with the non-treated control group. ^**^P < 0.01 and ^***^P < 0.001 compared to the Sin-1 or UVB-exposed group.

Although the IC_50_ value of swertiajaponin is similar to that of kojic acid in the cell-free experiment, actual inhibition of melanogenesis is greater in the swertiajaponin-treated cells than the kojic acid-treated cells, indicating that the direct binding to tyrosinase may not be the only mechanism underlying swertiajponin-mediated inhibition of melanogenesis. It is well-documented that the biological functions of flavonoids are associated with their anti oxidative effects [13]. Therefore, we studied whether swertiajaponin regulates oxidative stress by cell-free reactive oxygen (ROS) and peroxynitrite (ONOO^-^) scavenging assays. Swertiajaponin exhibited strong ROS and ONOO^-^ scavenging activities comparable to Trolox and penicillamine, well-known ROS and ONOO^-^inhibitors, respectively (Figure 5A-5B). We further tested whether the anti oxidative activity of swertiajaponin was also shown in B16F10 cells post UVB exposure. UVB exposure significantly increased ROS and ONOO^-^ production, whereas swertiajaponin decreased them in a concentration-dependent manner, suggesting that swertiajaponin is an antioxidant (Figure 5C-5D).

### Swertiajaponin inhibits UVB-induced MAPK activation

**Figure 6:**
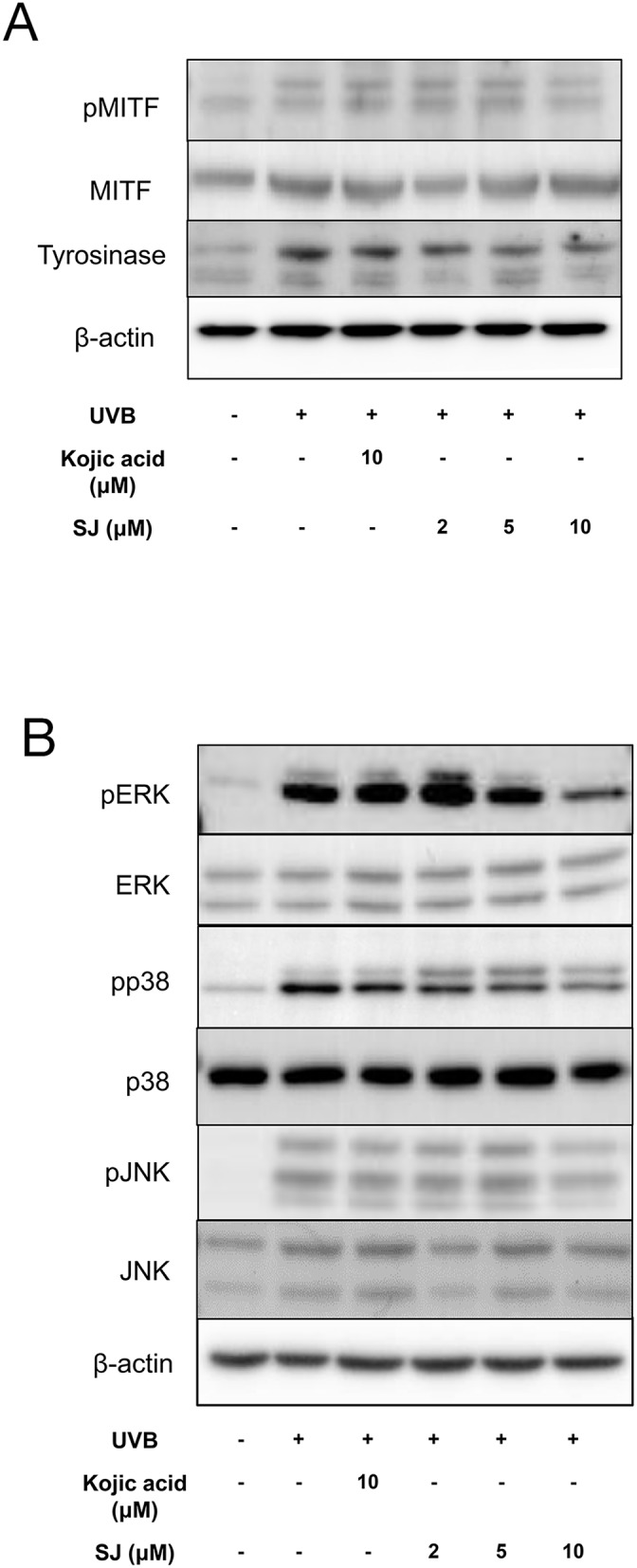
Swertiajaponin suppresses MAPK/MITF signaling in UVB-exposed B16F10 cells B16F10 cells were pretreated with swertiajaponin or kojic acid for 1 h and the cells were stimulated with UVB (10 mJ) for additional 6 h for MAPKs or 48 h for MITF and tyrosinase with swertiajaponin or kojic acid treatment (n=4/group). Western blotting was performed to detect protein levels of **(A)** pMITF, MITF and Tyrosinse, and **(B)** pERK, ERK, pp38, p38, pJNK and JNK in B16F10 cells. β-actin was used as the loading control.

Oxidative stress has been shown to stimulate melanogenesis by upregulation of microphthalmia-associated transcription factor (MITF), a transcription factor to induce tyrosinase gene expression [4, 14]. We studied whether the anti oxidative effect of swertiajaponin can regulate MITF activity. Western blotting data showed that UVB exposure increased phosphorylated MITF, an active form of MITF, whereas swertiajaponin treatment at 10 μM decreased it (Figure 6A). Consistently, a UVB-mediated increase in tyrosinase protein level was reduced by swertiajaponin treatment (Figure 6A). Because oxidative stress has been shown to activate MITF through mitogen-activated protein kinase (MAPK) [15, 16], it was investigated whether swertiajaponin can control MAPK signaling. UVB exposure dramatically increased phosphorylation of ERK, JNK, and p38 (Figure 6B), which is associated with the UVB-induced ROS and ONOO^-^ production in B16F10 cells (Figure 5C-5D). Swertiajaponin treatment only at 10 μM decreased the protein levels of MAPKs (Figure 6B). This result is consistent with the anti oxidative activity of swertiajaponin because the decrease in UVB-induced cellular oxidative stress was clear only at 10 μM of swertiajaponin (Figure 5C-5D).

Although swertiajaponin is the main compound of the whole herb of the Swertia japonica that has been used as a Japanese medicine, our study did not show conclusive evidence about the safety of swertiajaponin for its application to human skin. However, swertiajaponin did not exhibit cytotoxicity in cell lines of Hs27 (human fibroblast), HaCat (human keratinocyte), and B16F10 (mouse melanoma) in our experimental setting. Moreover, there were no visible signs of cytotoxicity including cell debris formation or cell detachment based on microscopic observation when swertiajaponin was treated to the human skin model with the concentrations that did not show cytotoxicity in the cell lines. Considering safety issues are main concerns of skin whitening compounds that are currently available, further *in vivo* studies are needed to examine its safety in physiology.

**Figure 7:**
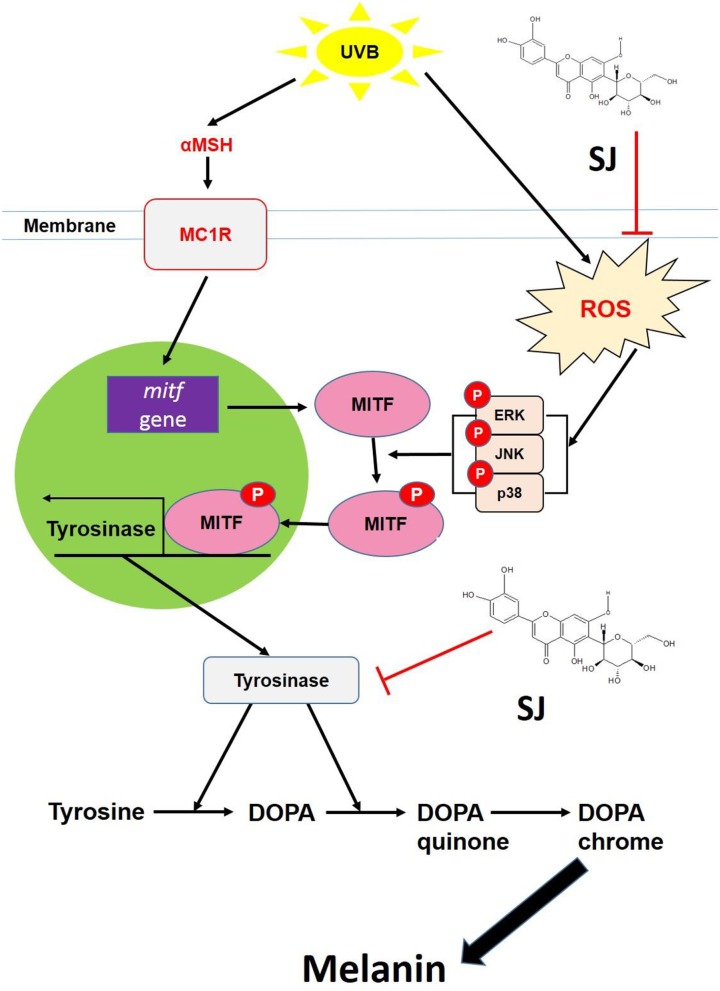
A hypothetical model of mechanisms underlying the swertiajaponin-mediated anti-melanogenic effect The images showed that swertiajaponin inhibits tyrosinase by direct binding to the active site of the enzyme and by the anti oxidative effect followed by suppression of MAPK/MITF signaling. Thus, it inhibits tyrosinase gene expression as well as its activity. MC1R, melanocortin 1 receptor.

Together, swertiajaponin inhibited melanin accumulation up to a satisfactory limit both in the cell and human skin models by dual mechanisms to suppress tyrosinase through direct binding to and competitively inhibiting tyrosinase and suppressing oxidative stress-mediated MAPK/MITF signaling (Figure 7). Considering the adverse effects and lack of long-term effectiveness of known skin whitening agents such as kojic acid and arbutin [17], swertiajaponin may be more safely applied to suppress skin pigmentation and would be a novel additive for whitening cosmetics.

## MATERIALS AND METHODS

### Tyrosinase activity assay using mushroom tyrosinase

Swertiajaponin and kojic acid (50 μM) were loaded into a 96-well microplate (Nunc, Denmark) in tyrosinase buffer (200 μL) containing mushroom tyrosinase (1000 U), 1 mM L-tyrosine solution, and 50 mM phosphate buffer (pH 6.5) [5]. The plate was incubated at 37 °C for 15 min and dopaquinone was evaluated by spectrophotometry (450 nm). Based on the measurement, the IC_50_ was calculated using log-linear curves and their equations.

### Docking simulation of swertiajaponin and tyrosinase

AutoDock Vina was used for the *in silico* protein–ligand docking simulation. The three-dimensional structure of tyrosinase was used in the crystal structure of *Agaricus bisporus* (PDB ID: 2Y9X). The predefined binding site of tyrosine was applied as a docking pocket. After docking simulations between tyrosinase and swertiajaponin or kojic acid were performed, the LigandScout 3.0 software was used to predict binding residues between different compounds and tyrosinase.

### Kinetic analysis of tyrosinase inhibition by swertiajaponin

L-DOPA was prepared at concentrations of 4, 2, 1, 0.5, 0.25, 0.125, and 0.0625 mM, and swertiajaponin was prepared at 20, 40, and 80 μM. Reaction mixture solution was prepared in a 96-well plate, in which 20 μL of tyrosinase substrate (L-DOPA), 10 μL of an aqueous mushroom tyrosinase solution (200 U), and 50 mM potassium phosphate buffer (pH 6.5) were added. The dopachrome production rate of the reaction mixture was measured at a wavelength of 450 nm using a microplate reader. The tyrosinase inhibition rate of swertiajaponin was then calculated using Lineweaver-Burk plot analysis. The Michaelis constant (K_m_) and maximal velocity (V_max_) were also calculated by Lineweaver-Burk plots with different concentrations of L-DOPA substrate [3].

### Cell culture and viability assay

B16F10 melanoma cells were purchased from the Korea Cell Line Bank. The cells were cultured in Dulbecco's modified Eagle's medium (DMEM) with 5% fetal bovine serum (FBS), and 1% penicillin, streptomycin, L-glutamine, and sodium pyruvate. The cells were maintained at 37 °C in a humidified 95% air/5% CO_2_ atmosphere. For the cell viability assay, cells were seeded in 96-well plates. B16F10 melanoma cells were treated with swertiajaponin at various concentrations for 48 h. Ez-Cytox (10 μL) was added to each well and incubated for 2 h. The formazan crystals formed was measured by spectrophotometry at 450 nm. Cell viability was calculated using cells without swertiajaponin treatment as the control group.

### Melanin content in B16F10 cells

Melanin level was measured using the method previously described with slight modifications [18]. B16F10 cells were allowed to grow to 70-80% confluence in 6-well plates. The cells were pre-treated with swertiajaponin or kojic acid for 2 h. Afterward, αMSH or UVB was treated to the swertiajaponin- or kojic acid-containing medium and incubated for further 48 h. After washing with PBS, the cells were detached using trypsin and dissolved in 90 μL 1 N NaOH solution containing DMSO (5%). After incubation at 60 °C for 1 h, melanin content was determined by measuring absorbance at 405 nm. A commercially available UV chamber (Boteck UV-X000, UV-AB, LAB24, korea) was used for the UVB exposure of B16F10 cells.

### Western blotting

Protein samples isolated from B16F10 cells (30 μg) were separated by sodium dodecyl sulfate-polyacrylamide gel electrophoresis using 10-12% acrylamide gels and transferred to polyvinylidene fluoride membranes, which were then immediately placed in blocking buffer (5% non-fat milk) in 10 mM Tris (pH 7.5), 100 mM NaCl, and 0.1% Tween 20. The membrane was washed in TBS-Tween buffer for 30 min and then incubated with specific primary antibodies (1: 1000 dilution) indicated in the figure legends at 4°C overnight. After washing with TBS-Tween buffer, the membrane was incubated with a horseradish peroxidase-conjugated anti-mouse antibody (Santa Cruz, 1 : 10,000), an anti-rabbit antibody (Santa Cruz, 1 : 10,000), or an anti-goat antibody (Santa Cruz, 1 : 10,000) at 25°C for 1 h. The immunoblots were visualized using Western Bright Peroxide solution (Advansta, CA, USA) and ChemiDoc Touch imaging system (Bio-Rad, U.S.A) according to the manufacturer's instructions. Antibodies used in this study are as following: p-CREB (Santa Cruz-81486), CREB (Santa Cruz-81486), tyrosinase (Cell Signaling-104976), pMITF (Abcam-59201), MITF (Abcam-20663), p-p38 (Cell Signaling-921L), p38 (Cell Signaling-9212S), pERK (Cell Signaling-4370L), ERK (Cell Signaling-9201L), pJNK (Cell Signaling-9251L), JNK (Cell Signaling-9252S), and β-actin (Santa Cruz-47778).

### Measurement of ROS and ONOO- levels

The antioxidative activity of swertiajaponin was examined using fluorescent 2,7-dichlorodihydrofluorescein diacetate (DCFDA) for ROS and dihydrorhodamine (DHR) 123 for ONOO-. Briefly, to determine ROS level, DCFDA (25μM) was added to cell homogenates for a final volume of 250μl. To measure ONOO- level, 10μl of cell homogenates was added to a rhodamine solution (50mM sodium phosphate buffer, 90mM sodium chloride, 5mM diethylenetriaminepentaacetic acid [DTPA], and DHR123). For both assays, fluorescence was measured every 5min for 30min on a fluorescence plate reader, with excitation and emission wavelengths set at 485 and 530 nm, respectively.

### Melanin accumulation in a human skin model

A viable, reconstituted, three-dimensional human epidermis (Neoderm-ME, Tego Science) was used to examine the anti-melanogenic effect of swertiajaponin in a human skin model. The human skin model was pretreated with DMSO (vehicle) or swertiajaponin for 1 h and cultured in the maintenance media provided by the company for 5 d with DMSO or swertiajaponin treatment. Microscopic analysis was performed at day 1 to day 5 to observe skin pigmentation. The microscopic images were analyzed by image J software to semi-quantify the darkening of the skin. For Fontana-Masson staining, skin samples were fixed in 4% paraformaldehyde overnight at room temperature and the samples were analyzed by a commercially available company (Garam Meditech, South Korea).

### Statistical analysis

All data are presented as mean ± SEM. Different groups were compared using one-way analysis of variance followed by the Dunnett's post test. *P* < 0.05 was considered statistically significant.

## SUPPLEMENTARY MATERIALS FIGURES


