# Supplementary material for: Swertiajaponin inhibits skin pigmentation by dual mechanisms to suppress tyrosinase
Source: Oncotarget. 2017 Sep 15;8(56):95530–41. doi: 10.18632/oncotarget.20913 (PMC5707040; doi:10.18632/oncotarget.20913)
Supplement: Supplementary file 1 [file oncotarget-08-95530-s001.pdf]

Swertiajaponin inhibits skin pigmentation by dual mechanisms to suppress tyrosinase

SUPPLEMENTARY MATERIALS

화합물번호	화합물명	화합물번호	화합물명
F1	(-)-Epicatechin	F26	Ononin
F2	Benzoic acid	F27	Poncirin
F3	Biochanin A	F28	Puerarin
F4	Calycosin-7-O-beta-glycoside	F29	Quercetin 3-β-D-glucoside
F5	Chrysin	F30	Quercitrin
F6	Cimifugin	F31	Rutin
F7	Daidzin	F32	Trifolirhizin
F8	Epimedin A	F33	Vitexin
F9	Epimedin B	F34	Wogonin
F10	(-)-Epigallocatechin gallate	F35	Wogonoside
F11	Epimedin C	F36	Orientin
F12	Eriodictyol-7-O-glucoside	F37	Baicalin
F13	Formononetin	F38	Iso vitexin
F14	Genistein	F39	Swertisin
F15	Genistin	F40	Swertiajaponin
F16	Hesperidin	F41	Casticin (Vitexicarpin)
F17	Hyperoside	F42	(-)-Liquritigenin Radix glycyrrhizae
F18	Icariin	F43	Eriodictyol
F19	(+)-Catechin Hydrate	F44	Dihydrokaempferol
F20	Isoliquirtin	F45	Baicalin hydrate
F21	Calycosin	F46	(±)-Dihydrokaempferol
F22	Liquiritin	F47	Bavachin
F23	7,3',4'5'-Tetramethoxyflavanone	F48	Warfarin
F24	Naringin	F49	Galangin
F25	Neohesperidin	F50	(+)-catechin

Supplementary Figure 1: The flavonotds used in the tyrosinase activity assay.
